# Association between serotonin transporter gene polymorphisms and increased suicidal risk among HIV positive patients in Uganda

**DOI:** 10.1186/s12863-017-0538-y

**Published:** 2017-07-25

**Authors:** Allan Kalungi, Soraya Seedat, Sian M. J. Hemmings, Lize van der Merwe, Moses L. Joloba, Ann Nanteza, Noeline Nakassujja, Harriet Birabwa, Jennifer Serwanga, Pontiano Kaleebu, Eugene Kinyanda

**Affiliations:** 1 Department of Biotechnical and Diagnostic Sciences, College of Veterinary Medicine, Animal Resources and Biosafety (COVAB), Makerere University, Kampala, Uganda; 2 Mental Health Project, Medical Research Council/Uganda Virus Research Institute (MRC/UVRI) Research Unit on AIDS, Kampala, Uganda; 3 Department of Psychiatry, Stellenbosch University, Cape Town, South Africa; 4 Division of Molecular Biology and Human Genetics, Faculty of Health Sciences, University of Stellenbosch, Cape Town, South Africa; 5 Department of Statistics, University of the Western Cape, Cape Town, South Africa; 6 School of Biomedical Sciences, College of Health Sciences, Makerere University, Kampala, Uganda; 7 Department of Psychiatry, College of Health Sciences, Makerere University, Kampala, Uganda; 8 Butabika National Psychiatric Referral Hospital, Kampala, Uganda; 9 Basic Science Programme, MRC/UVRI Uganda Research Unit on AIDS, Kampala, Uganda; 10 Faculty of Epidemiology & Population Health, London School of Hygiene & Tropical Medicine, London, UK

**Keywords:** Suicidal risk, Serotonin transporter (5-HTT) gene polymorphisms, HIV/Aids, Uganda

## Abstract

**Background:**

Persons living with HIV/AIDS (PLWHA) are at an increased risk of suicide. Increased suicidal risk is a predictor of future attempted and completed suicides and has been associated with poor quality of life and poor adherence with antiretroviral therapy. Clinical risk factors have low predictive value for suicide, hence the interest in potential neurobiological correlates and specific heritable markers of suicide vulnerability. The serotonin transporter gene has previously been implicated in the aetiology of increased suicidal risk in non-HIV infected study populations and its variations may provide a platform for identifying genetic risk for suicidality among PLWHA. The present cross-sectional study aimed at identifying two common genetic variants of the serotonin transporter gene and their association with increased suicidal risk among human immunodeficiency virus (HIV)-positive adults in Uganda.

**Results:**

The prevalence of increased suicidal risk (defined as moderate to high risk suicidality on the suicidality module of the Mini Neuropsychiatric Interview (M.I.N.I) was 3.3% (95% CI, 2.0–5.3). The *5-HTTLPR* was found to be associated with increased suicidal risk before Bonferroni correction (*p*-value = 0.0174). A protective effect on increased suicidal risk was found for the *5-HTTLPR*/rs*25531 S*_*A*_ allele (*p*-value = 0.0046)- which directs reduced expression of the serotonin transporter gene (*5-HTT*).

**Conclusion:**

The *S*_*A*_ allele at the *5-HTTLPR/*rs*25531* locus is associated with increased suicidal risk among Ugandan PLWHA. Further studies are needed to validate this finding in Ugandan and other sub-Saharan samples.

## Background

HIV/AIDS is associated with a considerable risk of suicide with studies reporting rates of between 7.8% to 43%, [1–5]. Increased suicidal risk is a predictor of future attempted and completed suicides, and has been associated with poor quality of life and poor adherence with antiretroviral therapy [6–8]. While considerable work has been undertaken to understand psychosocial and clinical risk factors for suicidal risk, these have low predictive value for suicide, hence the interest in potential neurobiological correlates and specific heritable markers of suicide vulnerability [1, 2, 9]. Evidence from genetic and epidemiological studies suggests that genes play an important role in the predisposition to suicidal behavior [10, 11]. There is also evidence that suicidal behavior may aggregate in families independently of the familial transmission of major depression [12], suggesting the existence of independent genetic risk factors for suicidal behavior [13]. A disturbance in serotonin (5-HT) transmission is the most frequently reported neurobiological abnormality associated with suicidal behavior [14, 15].

The serotonin transporter (5-HTT) is known to influence serotonergic transmission by regulating the duration of serotonin in the synaptic cleft. The gene encoding 5-HTT (*5-HTT*) is located on chromosome 17 and is composed of 14 exons [16]. Several 5*-HTT* polymorphisms have been identified [17]. Two of the most commonly studied *5-HTT* polymorphisms are an insertion/deletion polymorphism located 1.2 kb upstream of the transcription initiation site of the *5-HTT*-linked polymorphic region (*5-HTTLPR)* and a variable number of tandem repeat polymorphism, located in the second intron of the gene [18] and abbreviated as *STin2*.VNTRs﻿. The *5-HTTLPR* polymorphism has been widely investigated for its role in increased suicidal risk and in other psychiatric disorders, mainly in western populations. [19, 20]. This polymorphism comprises two main alleles based on the presence or absence of a 44 base pair fragment. The first is a 14-repeat short (*S*) variant with less transcriptional activity and lower serotonin uptake from synaptic clefts and the second is the 16-repeat long (*L*) variant which has been found to possess more transcriptional activity [20]. In addition, Lesch et al. [9] found the *S*-allele to be significantly associated with reduced *5-HTT* binding in the brain, and lower *5-HTT* mRNA expression and 5- HT uptake in lymphoblasts, relative to the *LL* genotype [20]. A meta-analysis found a significant association between the *5-HTTLPR S* allele and suicidal behavior [21]. The *S* allele was also found to increase the risk of developing a depressive episode, and suicidal ideation following exposure to stressful life events, early adverse environments, maltreatment and childhood physical and sexual abuse [19, 22–24].

More recently, a polymorphism has been reported in the *5-HTT* gene on human chromosome 17, designated as rs*25531* which is an *A* to *G* substitution [25]. The presence of *A* or *G* allele results in *L*_*A*_, *S*_*A*_, *L*_*G*_ and *S*_*G*_ alleles. *L*_*A*_ and *S*_*A*_ alleles are associated with increased transcriptional activity of the *5-HTT* gene and are thus referred to as “overexpressing” alleles, while *L*_*G*_ and *S*_*G*_ are associated with reduced transcriptional activity of the *5-HTT*, referred to as “low-expressing” alleles. A study by Roy et al. 2007 [26] found that the low-expressing alleles (*L*_*G*_ and *S*_*G*_) increased the risk of suicidal behavior in male African-American substance dependent patients exposed to childhood trauma.

The *STin2* VNTR is found in intron 2 and consists of multiple repeat copies of a 16–17 bp element [27]. Three major alleles have been described, containing 9 (*STin 2.9*), 10 (*STin 2.10*) and 12 (*STin 2.12*) repeats of the 16–17 bp repetitive element. These *STin2* VNTRs have been found to support differential gene expression in vitro [20, 28, 29]. For example, Lovejoy et al. [28] demonstrated that the 9, 10 and 12 repeat elements within the VNTR domain yielded extremely high levels of enhancer activity relative to the cytomegalovirus supported control, with the 9-repeat allele exhibiting greater enhancer activity in an embryonic stem cell model. In addition, Ali et al. [30] reported on an interaction between the *STin2* VNTR and 5*-HTTLPR* to regulate expression of the *5-HTT*. They reported that the *STin2.10* and *STin2.12* variants, which alone did not support additional activity, in conjunction with the *S-*allele (*S10* and *S12*) directed higher transcriptional activity of the *5-HTT*. However, no study has examined this polymorphism and increased suicidal risk in the context of HIV/AIDS. In addition, the *STin2* variant has not been widely studied in suicidal behavior. Only a few studies have shown a significant association between polymorphisms at this site and suicidal behavior [31].

There is a paucity of data from sub-Saharan Africa on genetic risk factors for increased suicidal risk. Additionally, globally, there is an absence of studies on genetic risk factors for suicidality among patients living with HIV/AIDS. To address these gaps we undertook a study to investigate the genetic risk factors associated with particular suicidal behavior phenotypes in the sub-Saharan African setting of Uganda among persons living with HIV/AIDS. We hypothesized that the overexpressing alleles of the serotonin transporter gene variants would confer increased suicidal risk to the bearer.

## Methods

### Study design

This cross-sectional, genetics sub-study was part of a bigger EDCTP funded project that primarily investigated risk factors for psychiatric disorders among adults with HIV/AIDS in Uganda [32]. All consenting eligible HIV-infected patients attending the study health facilities during the specified study period were enrolled until the required sample size (*N* = 600) was attained. To be eligible, study participants had to be registered at the participating HIV clinics, not on anti-retroviral therapy (ART), 18 years or older, fluent in English or Luganda (the local language into which the study instruments were translated), and physically and mentally well enough to complete the interview (not suffering from a severe physical and mental disorder to require immediate medical and psychiatric attention). Participants who had defaulted on their most recent clinic visit were excluded.

### Participants

Study participants were recruited by the parent study from two HIV clinics run by The AIDS Support Organisation (TASO) at Entebbe hospital (semi-urban site) and Masaka hospital (rural site) between 6th May 2010 and 30th October 2012. Study participants completed a structured interview (undertaken by trained psychiatric nurses) that included, amongst others, a socio-demographic proforma and the suicidality module of the Mini International Neuropsychiatric Interview (M.I.N.I) [33]. Increased risk for suicide was defined as moderate to high risk suicidality, as per the suicidality module of the M.I.N.I. This structured interview, including the MINI suicidality module, was administered in the local language of Luganda (main language spoken in central and southwestern Uganda). The MINI has previously been translated into the local Luganda language using a process of forward and backward translation by teams of mental health professionals who were independent of each other, with a consensus meeting held to resolve any discrepancies [32]. Blood specimens (5 ml) were obtained via venipuncture into EDTA tubes, aliquoted and stored for the subsequent genetics and immunological analyses.

### Genotyping

Genomic DNA was extracted from whole blood samples using the QIAamp DNA Blood Mini Kit (Manchester, United Kingdom). Polymerase chain reactions (PCR) was carried out in 25 μl reaction volumes containing between 20 and 205 ng template DNA, 200 μM dNTP (Kapa Biosystems, Cape Town, South Africa), 5 μl of 10X *Taq* DNA polymerase buffer (Kapa Biosystems, Cape Town, South Africa), 1.0 mM magnesium chloride (Kapa Biosystems, Cape Town, SA), 0.625 units (U) Taq DNA polymerase (Kapa Biosystems, Cape Town, SA), and 0.5 μM of each primer, with bi-distilled water. All PCR-amplification reactions were performed in a GeneAmp PCR System 9700 (Perkin Elmer Biosystems, Foster City, CA, USA).

For the *5-HTTLPR/*rs25531 polymorphism, an initial denaturation step was performed at 95 °C for 3 min. Thereafter, a denaturation step was performed at 95 °C for 15 s (s), followed by the primer annealing step, at 60 °C for 15 s, and an elongation step, performed at 72 °C for 15 s. A final elongation step, at 72 °C for 10 min, was then performed. The denaturation and extension steps were repeated for 35 cycles using 5′ -FAM-*ATGCCAGCACCTAACCCCTAATGT*3’ and 5′-*GGACCGCAAGGTGGGCGGGA*3’ forward and reverse primers respectively that were adapted from Voyiaziakis et al. [34]. After amplification, products were electrophoresed on 2.0% agarose gels, in sodium borate buffer at 120 V (V) for about 40 min, using GelStar (KapaBiosystems, Cape Town, SA) stain, with the *L-* and *S-*alleles resulting in fragments of 419 bp and 375 bp, respectively. In order to discriminate between the rs*25531 A* and *G* alleles, 5 μl of the remaining amplicon was digested using 5 U *Msp*I restriction endonuclease (New England Biolabs, United Kingdom) in a 10 μl reaction overnight at 37 °C, 5ul of the digested product was subjected to capillary electrophoresis on the ABI 3130 XL Genetic Analyzer (Applied Biosystems). Fragment sizes of the alleles at the *5-HTTLPR/*rs25531 locus were as follows: *S*_*A*_ */S*_*A*_ = 281 bp; *L*_*A*_ */L*_*A*_ = 325 bp; *S*_*G*_ */S*_*G*_ *, L*_*G*_ */L*_*G*_ *, L*_*G*_ */S*_*G*_ = 151 bp; *L*_*G*_ */S*_*A*_ = 151 bp + 281 bp and *L*_*A*_ */S*_*G*_, *L*_*A*_ */L*_*G*_ = 325 bp + 151 bp.

For the *STin2* VNTR polymorphism, an initial denaturation step was performed at 95 °C for 2 min. Thereafter, a denaturation step was performed at 95 °C for 30 s (s), followed by the primer annealing step, at 60 °C for 30s, and an elongation step, performed at 72 °C for 30s. A final elongation step, at 72 °C for 5 min, was then performed. The denaturation and extension steps were repeated for 35 cycles using 5′-HEX-*GTCAGTATCACAGGCTGCGAG* 3′ and 5′ *TGTTCCTAGTCTTACGCCAGTG* 3′ forward and reverse primers respectively that were adapted from Battersby and colleagues [35]. The PCR products were run on a 1.5% agarose gel in order to determine the success of the PCR, before the samples were loaded on the ABI analyser.

### Sample preparation and analysis on the ABI prism

The loci containing the *5-HTTLPR*/*rs*25531, and *STin2* VNTR polymorphisms were amplified individually. Following agarose electrophoresis to determine success of each PCR, the amplicons and the digested products were combined in a 1:1 ratio for allele-specific size discrimination analysis, which was performed on the ABI 3130 XL Genetic Analyzer (Applied Biosystems, Foster City, United States of America). The multiplexed samples were then sent to the Central Analytical Facilities (CAF) laboratory at Stellenbosch University, where analysis on the ABI prism was done.

### Statistical analysis

Functions from R, a language and environment for statistical computing, and R packages *genetics*, *haplo.stats* and *MASS*, were used for all statistical analyses. R is freely available from URL http://www.R-project.org. Specifically, functions from *genetics* were used to derive genotype and allelic distributions and to test the Hardy-Weinberg Equilibrium (HWE), while allelic combinations and their frequencies were inferred with functions from *haplo.stats*.

Logistic regression was used to model the different definitions of increased suicidal risk susceptibility (dichotomous outcome: increased suicidal risk defined as either *lifetime* attempted suicide or *past month* attempted suicide) and ordinal logistic regression (function *polr* from R package *MASS* to model the 3 categories of increased suicidal risk (low, moderate and high risk), as defined by the suicidal risk assessment criteria of the suicidal risk module of the M.I.N.I. These models allowed us to control for potential confounders (study site, socio-economic status and major depressive disorder).

*P*-values for tests of association between the serotonin transporter gene polymorphisms and increased suicidal risk were generated. Firstly, these were obtained as nominal *p*-values without Bonferroni correction (α = 0.05). To perform a Bonferroni correction, the *p*-value threshold of 0.05 was divided by 9 (number of separate tests) to yield a corrected threshold *p*-value of 0.0056 (see Table 4).

### Ethical considerations

Ethics approval was obtained from the Uganda Virus Research Institute Science and Ethical Committee *(Ref # GC/127/11/08/04)* and the Uganda National Council of Science and Technology *(Ref# HS 1053)*. All study participants provided informed consent to participate in the study and for a blood specimen to be withdrawn from them for the genetics analyses. Study participants who were diagnosed with significant psychiatric problems were referred to mental health units at Entebbe and Masaka government hospitals and their care clinicians were notified and follow-up care was arranged.

## Results

Socio-demographic and clinical variables are shown in Table 1.

**Table 1:** Counts (%) of the characteristics of the study group

Characteristic	Count (%)
Site	
Entebbe	214 (39)
Masaka	341 (61)
Sex	
Male	131 (24)
Female	424 (76)
Education Level	
Primary or less	406 (73)
Secondary and above	147 (27)
Marital Status	
Married	274 (50)
Not married	279 (50)
Employment Status	
Farmer	168 (31)
Not a farmer	382 (69)
Duration of awareness of HIV status	
< 12 months	145 (26)
12 months or more	407 (74)
Religion	
Catholic	306 (55)
Protestant	107 (19)
Other	142 (26)

Other: Muslims, Born-again Christians, Seventh Day Adventists etc. combined

### Prevalence of increased suicidal risk

The prevalence of increased suicidal risk, according to the criteria for ‘moderate to high suicidal risk’ on the M.I.N.I., was 3.3% (95% CI, 2.0–5.3), with a lifetime rate of attempted suicide of 6.3% (95% CI, 4.5–8.8) and a past month rate of attempted suicide of 2.2% (95% CI, 1.2 to 4.0) (Table 2).

**Table 2:** The prevalence of increased suicidal risk as defined by three criteria of increased suicidal risk

Increased suicidal risk	Prevalence	Per 100 (95% CI)
Attempted suicide life-time	35 of 553	6.3 (4.5 to 8.8)
Attempted suicide past month	12 of 537	2.2 (1.2 to 4.0)
No, Low, Moderate and High suicidal risk (according to MINI)	18 of 547	3.3 (2.0 to 5.3)

*CI*. Confidence Interval, *MINI*, Mini International Neuropsychiatric Interview

### Distribution of the alleles and genotypes for the *5-HTTLPR*, *5-HTTLPR/*rs25531 and *STin2.*VNTR

The allele and genotype distributions of *5-HTTLPR*, *5-HTTLPR/*rs25531 and *STin2* VNTR are presented in Table 3.

**Table 3:** Allele and genotype distribution for 5-HTTLPR, 5-HTTLPR and STin2.VNTRs polymorphisms of the serotonin transporter gene

*5-HTTLPR*	
Genotype distribution:	Count (Frequency)
*L/L*	358 (0.65)
*L/S*	155 (0.28)
*S/S*	38 (0.07)
*5-HTTLPR/rs25531*	
Genotype distribution	Count (Frequency)
*L*_*A*_ */L*_*A*_	223 (0.40)
*L*_*A*_ */S*_*A*_	121 (0.22)
*L*_*A*_ */L*_*G*_	116 (0.21)
*L*_*A*_ */S*_*G*_	3 (0.01)
*S*_*A*_ */S*_*A*_	34 (0.06)
*S*_*A*_ */L*_*G*_	32 (0.06)
*L*_*G*_ */L*_*G*_	23 (0.04)
*L*_*G*_ */S*_*G*_	1 (0.00)
*S*_*G*_ */S*_*G*_	4 (0.01)
*STin2. VNTRs*	
Genotype distribution	Count (Frequency)
*12/12*	317 (0.57)
*12/10*	182 (0.33)
*10/10*	55 (0.10)

*5-HTTLPR*, serotonin transporter linked polymorphic region; *L*, long allele; *S*, short allele; *L/L*, long long; *L/S*, long short and *S/S*, short short genotypes; *L*_*A*_, and *S*_*A*_ are over expressing alleles while *L*_*G*_ and *S*_*G*_ are under expressing alleles of the *5-HTTLPR/rs25531* locus; *STin2*, Serotonin transporter intron 2; *VNTRs*, Variable number of tandem repeats; *10, 10 repeat VNTRs* and *12, 12 repeat VNTRs* for *STin2 VNTRS*

The genotype distribution at the *STin2* VNTR and *5-HTTLPR* deviated significantly from HWE (*p* = 0.0036 and *p* = 0.0054 respectively) (Table 4).

The estimated effect of the *L*_*A*_ allele was to increase the odds of an individual being in a higher suicide risk category (from no to low, or low to moderate) by 53% (95% CI: 12% to 112% (*p* = 0.0068) before Bonferroni correction while the addition of an *S*_*A*_ allele reduced the odds of an individual being in a higher suicide risk category by 43% (95% CI: 14 to 63% (*p* = 0.0046) after Bonferroni correction.

There was also a significant association of the genotypes *L*_*A*_ *L*_*A*_, *L*_*A*_ */S*_*A*_, *L*_*A*_ */L*_*G*_ and *L*_*A*_ */S*_*G*_ (representing genotypes found to be associated with increased expression of *5-HTT*) versus the genotypes *S*_*A*_ */S*_*A*_ *, S*_*A*_ */L*_*G*_ *, L*_*G*_ */L*_*G*_ *, L*_*G*_ */S*_*G*_ and *S*_*G*_ */S*_*G*_ (representing genotypes found to be associated with reduced expression of *5-HTT*) with increased suicidal risk outcome (*p*-value 0.0145). However this association became non-significant after Bonferroni correction (Table 4). For the *STin2* VNTR, the rare 9-repeat allele was absent in the sample.

**Table 4:** Association between the 5-HTTLPR, rs25531 and STin2. VNTR and increased suicidal risk

Polymorphism/ allele	Suicide risk categories			
	No Bonferroni correction (α = 0.05)		With Bonferroni correction (α =0.0056)	
	Genotype	Allelic	Genotype	Allelic
*STin2.VNTRs*		*0.0285		0.0285
*5-HTTLPR*		*0.0174		0.0174
*rs25531*		0.6125		0.6125
*5-HTTLPR-rs25531*	0.0732		0.0732	
*L*_*A*_		*0.0068		0.0068
*S*_*A*_		*0.0046		*0.0046
*L*_*G*_		0.3491		0.3491
*S*_*G*_		0.5686		0.5686
Func_Comb	*0.0145		0.0145	

*p* values were corrected for study site, socio-economic status and major depressive disorder

*Significant effect, Func_Comb = Functional combination (over expressing (*L*_*A*_, *S*_*A*_) vs lower expressing (*L*_*G*_, *S*_*G*_ alleles). The STin2.VNTRs, serotonin transporter intron 2 variable number of tandem repeats, rs25531, *A* to *G* single nucleotide polymorphism at position 25,531 of the serotonin transporter gene, *5-HTTLPR-rs25531*, Serotonin transporter linked polymorphic region –*rs25531* haplotype

## Discussion

In this study, we investigated the genetic risk for increased suicidality among HIV positive persons in Uganda. To our knowledge, this is the first sub-Saharan African study to investigate the association of polymorphisms in *5-HTT* and increased suicidal risk, and the first such study in the world to investigate this risk among persons living with HIV/AIDS. In this study, the prevalence of moderate to high risk suicidality, according to the MINI, was 2.2%. Kinyanda and colleagues, 2012a [1], using a similar methodology in a study undertaken at government run HIV clinics at the semi-urban site of Entebbe, reported a prevalence of increased suicidal risk of 7.8% and lifetime attempted suicide of 3.9%. [1]. Rukundo and colleagues (2016), in a semi-urban HIV clinic in south-western Uganda and using a different method for assessing suicidal risk, reported a prevalence of suicidal ideation of 8.8% and lifetime attempted suicide of 3.1% [2]. These rates from Uganda are, however, much lower than those reported elsewhere: 26% for suicide ideation in the USA [3], 43% for suicidal ideation in China [4], 31% in United Kingdom [7], 21% in Australia [36] and 26.9% in Europe and America (from a systematic review) [5]. The reasons for these differences include diversity of assessment methods for suicide risk and differences in underlying suicide risk associated with particular HIV risk categories (in HIV endemic countries like Uganda the HIV risk category is the general population while in the west and in China the HIV risk categories are men who have sex with men and intravenous drug users). For a complete discussion, see Kinyanda and colleagues 2012a [1].

A recent review provided evidence for an association between *5-HTT* and suicidal behavior [13]. *5-HTT* has been a focus of investigation in affective disorders. A better understanding of these genetic variants and others that control *5-HTT* function (including non-*5-HTT* sources of epistasis) will be important in predicting the effects of *5-HTT* variation on a variety of emotional phenotypes associated with increased suicidal risk [37].

In the brain, impulses are passed from one nerve cell to another via a synapse. The pre-synaptic cell releases serotonin (5-HT) into the synaptic cleft, where it interacts with both post- and presynaptic receptors. At the presynaptic side, 5-HT activates 5-hydroxytryptamine (serotonin) receptor 1A (HTR1A), B (HTR1B), and D (HTR1D), which in turn relays the signal [38]. About 90% of 5-HT is sent back to the pre-synaptic cell. 5-HTT clears 5-HT from the synaptic clefts, regulating the strength and duration of serotonergic signaling. Ho et al., [39] observed a significant difference in 5-HTT availability in the thalamus (measured by positron emission tomography 5-HTT imaging technique) in major depression patients and healthy controls. Overexpression of *5-HTT* leads to more serotonin transporter in the synaptic cleft [17, 20] and increases the efficiency of serotonin transportation back into the pre-synaptic cell resulting in serotonin not to linger at the synapse. This consequently leads to a shorter action of serotonin. Reduced serotonin activity is thus expected to be found in the neurons of individuals with the *L*_*A*_ allele. These individuals are probably at an increased risk of developing major depression, as dysfunction of serotonin neurotransmission has been associated with occurrence of major depressive disorder [40]. As depression has been found to be an independent predictor of moderate-to-high suicidal risk among ambulatory HIV patients in Uganda [1], we hypothesized that the overexpressing alleles would be the risk alleles for increased suicidal risk. A nominally significant association between the *5-HTTLPR* and increased suicidal risk (*p*-value = 0.0174) was found with the *S*-allele being protective and the *L*-allele being a risk factor for suicidality. Previous studies of the *5-HTTLPR* have found the *S*-allele to be a risk factor for increased suicidality [41], while others have found no significant association [42]. The discrepancy in findings may be related to sample size, sample ethnicity or other confounders such as sampling methods. Thus proper control for confounders is critical when replicating studies.

The *5-HTTLPR*-rs25531 *L*_*A*_ allele was found to be associated with an increased risk of suicidality (*p*-value = 0.0068), with a 53% increase in the odds of an individual being in a higher suicide risk category (from no-to-low, or low-to-moderate risk) if they carry the *L*_*A*_ allele (95% CI: 12% to 112%), however this association became less significant after Bonferroni correction. The *L*_*G*_ and *S*_*G*_ alleles were not significantly associated with increased suicidal risk (*p*-values = 0.3491 and 0.5686 respectively), while individuals possessing the *S*_*A*_ allele were found to have a 43% (95% CI: 14 to 63%) reduction in odds of increased suicidal risk (*p*-value = 0.0046).

Our results are somewhat in line with those from previous studies. Henriette et al. [43] found a statistically significant protective effect of the *5-HTTLPR S*-allele for suicide among individuals below 35 years of age with a contrasting statistically significant protective effect of the high expression genotype (*L*-allele) for individuals between 35 and 49 years in a Danish sample. We did not stratify for age, thus the significance of age suggested by Henriette was not tested for. In addition, Goldman et al., 2010 [44] observed low rates of depression, despite a high frequency of the *S-*allele, among an East Asian population. Also, among Caucasians of American descent, those with the *S/S* genotype had lower 5-HIAA levels than those with either *L/L* or *L/S* genotypes [45]. These findings seem to suggest that the *S-*allele is protective against depression in African-Americans and East-Asian populations, unlike in Caucasian populations, where it acts as a risk allele. The discrepancy amongst these different populations could be due to linkage disequilibrium between the *S-* allele and other *5-HTT* polymorphisms, such as the functional single nucleotide polymorphisms (SNPs) at rs*25532* and rs*6355* [37], that may result in a reversal of the effect of the *S/S* genotype on serotonin turnover among Africans or African-Americans which may be absent in Caucasian populations.

The association of *5-HTTLPR* with increased suicidal risk has been contradictory across various ethnicities. However, a recent review by Schild et al. [46] has reported a protective role of the *S* allele in Caucasian populations, whilst acting as a risk factor in non-Caucasian populations. In the present study, the *S*_*A*_ allele was protective against increased suicidal risk, a finding similar to that observed among Caucasians [46]. The discrepancy between findings of Goldman et al. [46] and those of Schild et al. [46] may be due to the fact that the non-Caucasians referred to by Goldman et al. [44] were of East Asian origin. Extrapolating previous findings seems difficult for samples of African ethnicity since there is paucity of data in the field. The contradiction in findings among Caucasians, Asians and African-Americans/Africans seems to suggest differences in *5-HTT* risk alleles and suicidality among different races, supporting the finding by Schild and colleagues [46] that ethnicity moderates the association between *5-HTTLPR* and national suicide rates.

A nominally significant association between the *STin2* VNTR and increased suicidal risk (*p*-value = 0.0285) was found in the present study. However, this association became non-significant after Bonferroni correction. The *STin2.12* allele increased the odds of an individual falling into a higher suicidal risk category group, while the *STin2.10* allele decreased these odds. A study by Bah and colleagues [47] found an association of the *STin2.12* allele with a history of suicide attempts, which concurs with our findings. However, a previous study by Lopez de Lara and colleagues [48] found a significant association of suicide completion with having at least a copy of the *STin2.10* allele. Another study by Lee and colleagues [31] found the *STin2.10* allele to be associated with suicidal behavior. Findings by Lee et al. [31] and Lopez de Lara et al. [48] are in agreement, whilst contradicting those of the present study, which are in agreement with the findings of Bah et al. [47]. Mackenzie and Quinn, [49] reported the *STin2.12* to possess greater transcription activity of the *5-HTT* than the *STin2.10*. The *STin2.12* could thus be compared to the overexpressing *L*_*A*_ allele whilst the *STin2.10* could be compared to the less expressing *S*_*A*_ allele. As per our hypothesis that over expressing alleles of the *5-HTT* confer increased suicidal risk to the bearer, the over expressing *L*_*A*_ allele of the *5-HTTLPR*/rs*25531* was found to be a risk allele while the lower expressing *S*_*A*_ allele was found to be protective against increased suicidal risk. Since the *STin2.12* compares with L_A_ and *STin2.10* compares with *S*_*A*_ in their capacity to direct transcription of the *5-HTT*, this is interesting, as the finding at the polymorphic intron 2 region replicates that at the *5-HTTLPR*.

Neither the *5-HTTLPR* nor the *STin2* VNTRs genotypes were in Hardy-Weinberg equilibrium (HWE). There exist a number of reasons for deviation from HWE, including selection bias and genotypic errors. In the present study, it is likely that deviation from HWE could result from selection bias. Participants were not randomly selected from the general population, but were all HIV+ adults who were recruited from HIV care clinics. Another cause of the Hardy-Weinberg Disequilibrium (HWD) could have been genotyping errors as genotyped samples were not re-genotyped in order to eliminate the presence of genotypic errors. However, genotyping errors as a cause of HWD is contentious - McCarthy and colleagues [50] reported, in hypothetical data, that the presence of HWE was generally not altered by the introduction of genotyping errors [51], thus ruling out genotypic errors as the possible cause of the HWD. Finally, the violation of the assumptions for HWE could have been due to population stratification or selection bias as discussed above. Future studies should endeavour to control for these when sampling.

This is first study to investigate the interactions between *5-HTTLPR* and *5-HTTLPR-*rs25531 polymorphisms, *STin2.*VNTR polymorphisms and increased suicidal risk in PLWHA.

### Limitations

Population stratification was not controlled for at analysis. We assumed that majority of our sample was of Bagandan ethnicity based on the language spoken (Luganda) and self report. There is a possibility that some participants were not Bagandan even though they could speak Luganda, indicating that we may have had an admixed sampled, resulting in violation of HWE. Future studies should focus on ethnicity using ancestry informative markers to delineate subjects genetically, rather than basing ethnic differentiation on language spoken and or self report.

The significant associations of the *STin2* VNTRs observed became non-significant when we controlled for multiple testing using the Bonferroni correction. It is possible that the significance observed for the above named polymorphisms may have been due to chance. Replication studies in the same population are, therefore, needed to confirm these findings.

## Conclusions

Both the *L*_*A*_ allele of the polymorphism at the *5-HTTLPR/rs25531* locus and the *12-repeat* of the polymorphism at STin2 region in the *5-HTT* are risk alleles for increased suicidal risk among PLWHA in a Ugandan population.

Results of the present study require replication in a larger study, which is more equipped to address population stratification, and provides more power for association analyses.

There is need for further genotyping studies in Ugandan samples to confirm these findings.

The majority of previous studies have investigated the *5-HTT* in Caucasian populations in Europe and America and more studies are needed in African populations.

## Abbreviations

5-HIAA: 5-hydroxy indole acetic acid;
5-HT: Serotonin
5-HTT: Serotonin transporter
5-*HTTLPR*: Serotonin transporter linked polymorphic region
CSF: Cerebral spinal fluid
DNA: Deoxyribonucleic acid
EDCTP: European Developing Countries Clinical Trials Partnership
HIV/Aids: Human Immunodeficiency Virus/ Acquired immunodeficiency syndrome
MINI: Mini International Neuropsychiatric Interview
PCR: Polymerase chain reaction
PLWHA: People living with HIV/Aids
SNPs: Single nucleotide polymorphisms
STin2: Serotonin transporter intron 2
TASO: The Aids Supporting Organisation
*VNTR*: Variable number tandem repeat
